# Evidence-Based Decision Aid for Patients With Parkinson Disease: Protocol for Interview Study, Online Survey, and Two Randomized Controlled Trials

**DOI:** 10.2196/17482

**Published:** 2020-07-14

**Authors:** Martina Bientzle, Joachim Kimmerle, Marie Eggeling, Idil Cebi, Daniel Weiss, Alireza Gharabaghi

**Affiliations:** 1 Leibniz-Institut für Wissensmedien Tübingen Germany; 2 Department of Psychology University of Tübingen Tübingen Germany; 3 Division of Functional and Restorative Neurosurgery and Tübingen NeuroCampus University of Tübingen Tübingen Germany; 4 Department of Neurodegenerative Diseases Hertie Institute for Clinical Brain Research University of Tübingen Tübingen Germany; 5 German Centre of Neurodegenerative Diseases Tübingen Germany

**Keywords:** decision aids, Parkinson disease, interview study, online survey, randomized controlled trial, patients

## Abstract

**Background:**

Shared decision making is particularly important in situations with different treatment alternatives. For the treatment of idiopathic Parkinson disease, both pharmacological and surgical approaches can be applied.

**Objective:**

In this research project, a series of studies will be conducted to investigate how decision aids for patients with idiopathic Parkinson disease should be designed in order to support the decision-making process.

**Methods:**

In Study 1a, qualitative interviews will be conducted to determine which needs frequently occur for patients with idiopathic Parkinson disease. In Study 1b, the identified needs will then be rated for personal relevance by an independent group of patients in an online survey. In Study 2, a randomized controlled trial will be used to pretest different decision aids in a sample group of people who do not have a medical background and who do not have Parkinson disease. In Study 3, a randomized controlled trial will be used to investigate the effect of the decision aids that had been evaluated as positive in Study 2 with patients who have idiopathic Parkinson disease.

**Results:**

This series of studies received ethical approval in January 2020. As of June 2020, data collection for Study 1a has started, and it is estimated that Studies 1a, 1b, 2, and 3 will take approximately 4, 4, 6, and 6 months to complete, respectively. It is planned to present the results and analyses at international conferences and to submit the results to peer-reviewed journals for publication, once the studies have been completed. The findings will also be shared with clinicians and patients through presentations at information events.

**Conclusions:**

This series of studies is intended to result in an evidence-based decision aid for patients with idiopathic Parkinson disease in order to support the informed and reflected shared decision-making process. We further intend to contribute to a deeper understanding of the individual preferences of patients with idiopathic Parkinson disease and the impact of those preferences on treatment decisions.

## Introduction

### Background

Shared decision making has become increasingly common in the context of medical consultations [1-3]. While in any medical consultation it is useful and ethically necessary to inform and educate a patient thoroughly, participatory decisions are particularly appropriate in situations where different alternatives need to be considered and for which no one treatment is superior to the others. In these situations, a preference-sensitive decision should be made based on the personal circumstances and individual preferences of the patient [4-6]. Research indicates that patient participation in the medical decision-making process has positive effects. A meta-analysis [7] with a total of 105 studies showed that shared decision making led to increased knowledge, higher confidence in decisions, and more active patient participation. Studies [7,8] found that the use of shared decision making could reduce health care system costs, because patients often chose less invasive (and therefore, less expensive) treatment options. In addition, most patients would like to be more involved in the medical decision-making process [9]. But, despite these promising findings, shared decision making has not yet been sufficiently implemented in clinical practice [10,11]. The Revised Program Theory for shared decision making [12] identified relevant factors influencing engagement in shared decision making. The authors of that paper [12] explicitly foster future research using this theory and examining additional key mechanisms of shared decision making. Based on these theoretical considerations, herein we will examine the influence of health care system support through decision aids.

### Treatment of Parkinson Disease

For the treatment of idiopathic Parkinson disease, both pharmacological and surgical methods can be used. The 2016 *Leitlinien für Diagnostik und Therapie in der Neurologie–Idiopathisches Parkinsonsyndrom* *(Guidelines for Diagnosis and Therapy in Neurology–Idiopathic Parkinson Disease)* of the *Arbeitsgemeinschaft der Wissenschaftlichen Medizinischen Fachgesellschaften (Association of the Scientific Medical Societies)* and the *Deutsche Gesellschaft für Neurologie (German Society of Neurology)* [13] recommended offering subthalamic nucleus–deep brain stimulation to patients with a confirmed diagnosis of idiopathic Parkinson disease who, despite best medical treatments, have motor fluctuations and dyskinesia that cannot be treated with medication, or tremor that cannot be controlled with medication (recommendation 47 in [13]). In addition, deep brain stimulation of the subthalamic nucleus can be offered to patients 60 years of age or younger with confirmed idiopathic Parkinson disease in the first three years after the onset of motor fluctuations or dyskinesia (recommendation 48 in [13]).

Furthermore, it was emphasized that when data suggest deep brain stimulation rather than best medical treatment, surgical treatment should remain an individual decision as long as medical alternatives exist [13]—even if the outcome of drug treatment may be worse than that of subthalamic nucleus–deep brain stimulation in such cases. Nevertheless, since subthalamic nucleus-deep brain stimulation may be more effective than pharmacological treatment, this surgical alternative should be discussed with the patient. When deciding between subthalamic nucleus–deep brain stimulation or drug therapy, patients should be involved in the decision-making process in order to be able to make a preference-sensitive decision with the physician (recommendation 66 in [13]).

### Shared Decision Making

Doctors in clinical practice often have too little time for lengthy conversations and tend to overestimate the understanding and health literacy of patients [14,15]. Low health literacy of patients with idiopathic Parkinson disease has been shown to correlate with an increased risk of hospitalization [16]. A meta-analysis showed that the perception of traditional role models, in which doctors are the experts whose instructions are followed, represents a barrier for many patients to actively participating in the decision-making process [17]. One way to deal with this barrier is to use decision aids. They are often used to provide information about illnesses and treatment options and have shown their value in various medical fields [18,19]. Decision guidance is an opportunity to support both patient education and informed decision-making in cooperation with attending physicians and therapists. It is explicitly emphasized that decision aids should not claim to replace patient to doctor direct contact or conversations. Rather, they should be viewed as a supplement that makes it easier to take individual needs into account. The guidelines [13] also recommended that patients with idiopathic Parkinson disease should be provided with individually adapted need-based enhanced communication throughout the course of their disease (recommendation 67 in [13]). According to the Revised Program Theory [12] of shared decision making, giving patients support for shared decision making (for example, by means of a well-designed decision aid) may result in higher confidence in their decision-making abilities and in stronger engagement in shared decision making. Studies [20-22] showed that many patients with Parkinson disease want to play a more active role in treatment decisions [20]. As one study [21] that interviewed patients with Parkinson disease showed, patients often had to take the initiative themselves to be referred to a deep brain stimulation center. Only 10% to 15% of patients for whom deep brain stimulation might be considered a suitable therapy option were, in fact, referred to specialist centers. This finding can be attributed, among other reasons, to insufficient information on the part of patients and neurologists alike [22].

### Decision Aids

There are many ways to support patients in their decision-making process and to inform them about the disease and its possible treatment alternatives. Experience reports (ie, narratives) from other patients are an important source of information in addition to exchanges with medical specialists. Patients exchange information about their situation with other people and use the internet in their search for medical information often finding testimonials from other patients [23]. Patient reports are also often integrated into decision-making aids. The use of narratives in decision aids is critically discussed in the literature [24-26]. Narratives have the advantage that they are vivid, easy to understand, and not too abstract, which can make it easier for patients to process and remember the information conveyed [24,27]; therefore, this format appears to be particularly suitable for patients with idiopathic Parkinson disease, as their cognitive abilities may also be limited. Entwistle et al [28] concluded, in a qualitative interview study, that the personal experiences of others combined with the imparting of factual knowledge can be very helpful for decision making. The patients who were interviewed stated that the reports helped them to better imagine the different options, to become clear about what was important to them personally, and to handle their negative emotions; however, narratives can also have the disadvantage of encouraging the activation of heuristics and generating additional emotions, which can lead to a distorted perception of the information given [25]. In a study [29] with a focus on persuasion, it was found that information mediation with narratives, when compared with scientific information mediation, led to more knowledge gained, more emotions, and stronger persuasion. Narratives also partly influenced the decisions of people who do not have a medical background [30]. In preference-sensitive decision situations, where the needs of the patient should be a key factor in the decision, the persuasive effect of decision support would be considered problematic. The decision-making process of patients should be supported in the preference-sensitive situations that occur in patients with idiopathic Parkinson disease, without convincing them of any one of the possible treatment options. According to the Expected Utility Theory [31,32], it is easier for patients to engage in shared decision making if outcome probabilities of a given treatment are presented. But in preference-sensitive situations, no treatment is superior to the other. As a result, decision difficulty in these situations would be perceived as rather high [12]. The question arises as to whether it makes sense in this situation to report clinical outcomes at all, or whether it is more useful to provide information about possible personal motives and preferences. Decision aids and narratives can be designed very differently and can exert various positive or negative influences on decision making as a result of their design. It is important to understand how decision aids should be designed to help patients take their knowledge, their personal preferences, and their needs into account when making decisions, without pushing them in any specific direction.

### Goals of the Research Project

In this research project, a series of studies will be conducted to investigate how decision aids for patients with idiopathic Parkinson disease should be designed in order to support the decision-making process. The goal is to support patients in taking their individual preferences into account when making a decision and to make them feel confident with their decision. We will use a participatory design process for the development of the decision aid [33]. Feedback and ideas for improvement will be requested from health care professionals before the prototype is used in the studies.

It is an open question whether the presentation of possible preferences has a positive influence on the decision-making process. In addition, it has not yet been clarified whether patient narratives can strengthen patient decisions in difficult decision situations. Moreover, we will act on the suggestion of the Revised Program Theory and examine additional key mechanisms of shared decision making. We aim to compare the impact of presenting motives that affect a decision with the impact of presenting treatment outcomes.

In Study 1a, qualitative interviews will be conducted to determine which needs frequently occur for patients with idiopathic Parkinson disease. In Study 1b, the identified needs will then be assessed for personal relevance by other patients in an online survey. In Study 2, a randomized controlled trial will be used to pretest different decision aids with people who do not have a medical background and who do not have Parkinson disease. In Study 3, a randomized controlled trial will be used to investigate the effect of the decision aids that were evaluated as positive in Study 2 in patients with idiopathic Parkinson disease.

## Methods

### Ethical Approval

These proposed studies have been reviewed and approved by the ethics committee of the Faculty of Medicine of the Eberhard Karls University Tübingen.

### Studies 1a and 1b: Expectations and Wishes Regarding Medical Treatment Options for Parkinson

#### Study Design

A qualitative interview study (Study 1a) with 6 patients with idiopathic Parkinson disease will identify the common needs, expectations, wishes, and preferences that play a role in the decision to opt for drug-only treatment over deep brain stimulation. Patients with idiopathic Parkinson disease who qualify for deep brain stimulation treatment according to recommendations 47 and 48 in [13] will be invited to a screening week as part of regular clinical care.

During this 1-week inpatient stay, the medical, cognitive, and psychological condition of patients will be examined in preparation for potential deep brain stimulation surgery. From a certain date onward, all patients who meet the inclusion criteria (see below) will be asked by the treating physician whether they agree to participate in a qualitative interview. A semistandardized interview lasting approximately 30 minutes will be conducted with those who agree to participate in order to identify the personal needs, hopes, fears, and expectations associated with drug treatment and deep brain stimulation treatment options. In an online survey study (Study 1b), a different group of patients with idiopathic Parkinson disease will rate the needs, hopes, fears, and expectations that were identified in Study 1a according to personal relevance. Patients with idiopathic Parkinson disease who are at different stages of their disease will be recruited for Study 1b. Patient assessment of their stage and burden of the disease will be measured using a validated questionnaire (Parkinson Disease Questionnaire, PDQ-39) [34].

#### Participants

In order to achieve a representative rating, a total of 60 patients will be recruited for the online survey study (Study 1b). Recruitment will be carried out with the support of the specialized outpatient units of the University Hospital Tübingen, neurologists in private practice, and self-help groups for patients with idiopathic Parkinson disease. Patients will be included if they have been diagnosed with Parkinson disease, have had Parkinson disease for at least 5 years, are between 18 and 80 years of age; have taken prescribed dopaminergic medication consistently for at least two weeks before inclusion in the study; have a very good knowledge of German, and have signed a written declaration of consent. Patients will be excluded if they have been diagnosed with dementia.

#### Study 1a: Interview Guide

During the interview, each area will be introduced with an open question (Textbox 1). Further questions will follow only if the answer is unclear or the question has not been answered.

**Open-ended questions and topics for semistructured interview.**

**Satisfaction with the current situation**

- How satisfied are you with the current situation? (scaling question: 0 = very dissatisfied, 100 = very satisfied)
- What should remain the same?
- What should change?

Other: mobility and motor skills, faculty of speech, sleep, memory and concentration ability, previous treatment, general state of health, execution of profession and hobbies, participation in social life

**Reasons and expectations**

- What are your main reasons for deep brain stimulation?
- What are your main reasons for a purely pharmacological therapy?
- What are other reasons?
- What should change?
- How would you know if the decision was good?
- In which area (eg, work, social life, hobbies) do you hope to have a positive influence?
- What are your plans for the time after the deep brain stimulation/drug changeover?
- What positive expectations/worries do you associate with a deep brain stimulation/drug changeover regarding your social environment, your self-reliance, your well-being?

#### Study 1b: Questionnaire

The needs, wishes, and worries that are identified in Study 1a will be evaluated in Study 1b. Patients who wish to participate will also have to confirm that they are currently in the process of deciding between further treatment with medication or deep brain stimulation. The questionnaire will initially present questions pertaining to inclusion and exclusion criteria, and if their responses indicate they are eligible, questions about personal needs, wishes, and worries will be provided.

A maximum of 20 statements such as (the following are example items only) “I hope to be able to maintain more personal contacts through a successful treatment”, “It is important to me that the treatment has as few side effects as possible,” and “I hope that the treatment will improve my independence” will be evaluated. In addition, participant knowledge about treatment options will be evaluated with maximum of 20 statements or questions such as (the following are example items only) “The danger that deep brain stimulation can lead to psychological impairment is very high” and “The danger that a purely pharmacological treatment can lead to psychological impairment is very high” where participants will indicate whether the statements are true or false and how confident they are in their answers:. Since patients with idiopathic Parkinson disease may display neuropsychiatric symptoms, such as apathy or emotional instability, the German version of the Apathy Evaluation Scale [35] and a German version of a short-scale for assessing the personality traits in the five-factor model (big five) [36] will be administered.

### Study 2: Effects of Decision Aids on the Decision-Making Process (People With Nonmedical Background)

#### Study Design

Based on the patient preferences identified in Study 1, online decision aids will be designed in close collaboration with health professionals. We will use a participatory design process to increase the later acceptance and use of the decision aids, and thus, facilitate the implementation process in clinical practice. The impact of the decision aids on the decision-making process will be investigated in a randomized controlled trial with a 1×3 between-subject design with the following 3 groups: condition 1, decision aid with factual information (control); condition 2, decision aid with factual information and patient reports on the individual motives underlying the decision-making process; and condition 3, decision aid with factual information and patient reports on the outcomes achieved through the treatment option.

In condition 3, positive and negative outcomes will be presented according to the actual success rate of the treatment options, that is, both successful and less successful treatment outcomes will be shown for both treatment options. The information provided to the participants regarding the success rates of the treatment options will be based upon existing literature in line with institutional history.

We hypothesize that the conditions containing patient reports on motives or outcomes will have a beneficial effect compared to the condition with only factual information; participants to whom decision aids with patient reports are presented will feel better prepared for decision making (Hypothesis 1) and will evaluate the decision more positively (Hypothesis 2). It is an open research question whether the two conditions with patient reports on individual motives and patient reports on outcomes will also differ with respect to preparation for decision making and decision evaluation.

In addition, this study will be used to evaluate and refine the factual information part of the decision aid. Participant knowledge about treatment options will be evaluated pre and posttest to identify which facts are difficult to understand (and which, perhaps, even result in misunderstandings or irrational fears). This information will influence the design of the factual information and will be re-evaluated in the subsequent study (Study 3).

#### Participants

Healthy individuals who are neither active in the medical field nor studying a medical subject will be recruited via the Leibniz Institut für Wissensmedien database of test subjects and the University of Tübingen email distribution list, where individuals who are not studying medical-related subjects can be preidentified. Participants at least 18 years old, not affected by Parkinson disease, and with a very good knowledge of German will be eligible for inclusion.

Since only medium to large effect sizes are relevant for our purposes, a sample size of 37 participants per condition will be targeted. The sample size was determined using G*Power [37] and is based on an alpha error probability of .05, a test power of .90, and an expected effect size of *f*=0.30 for an analysis of variance with repeated measurements and between-subject factors.

#### Procedure

After basic demographic data (age, gender, education) and individual prior experiences with Parkinson disease have been collected, the study participants will be asked to put themselves in the position of a patient with idiopathic Parkinson disease with the help of a case description. The following dependent variables will be measured as target variables before and after the use of the decision support: decisional conflict [38]; hypothetical decision (which treatment option would be preferred); attitude toward both treatment options (modified according to [39]); and knowledge about risks and side effects of treatment options, mode of action of treatment options, and possible advantages and disadvantages of treatment options.

Subsequently, participants will be randomly assigned (computer-generated assignments) to the 3 conditions. Based on the results of Study 1a and Study 1b, a text- and video-based decision aid will be designed with a maximum reception time of 15 minutes.

In addition, the following are to be completed after the decision aid has been used: preparation for decision making (based on [40]); evaluation of the preferences or motives identified in Study 1 with regard to the importance for the decision made, decision evaluation scale [41]; reflection on one’s own reasons for the decision; suggestions for improvement (open answer format), and the knowledge test.

#### Measures

**Figure 1:**
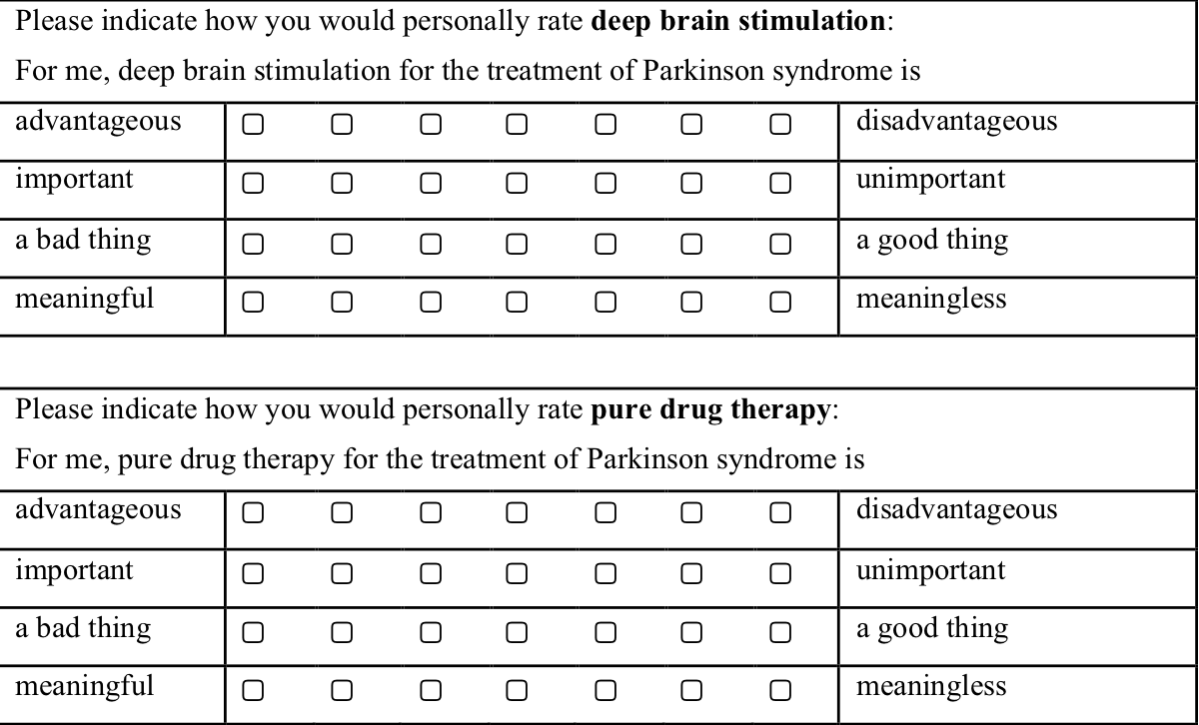
Attitude toward treatment options.

**Figure 2:**
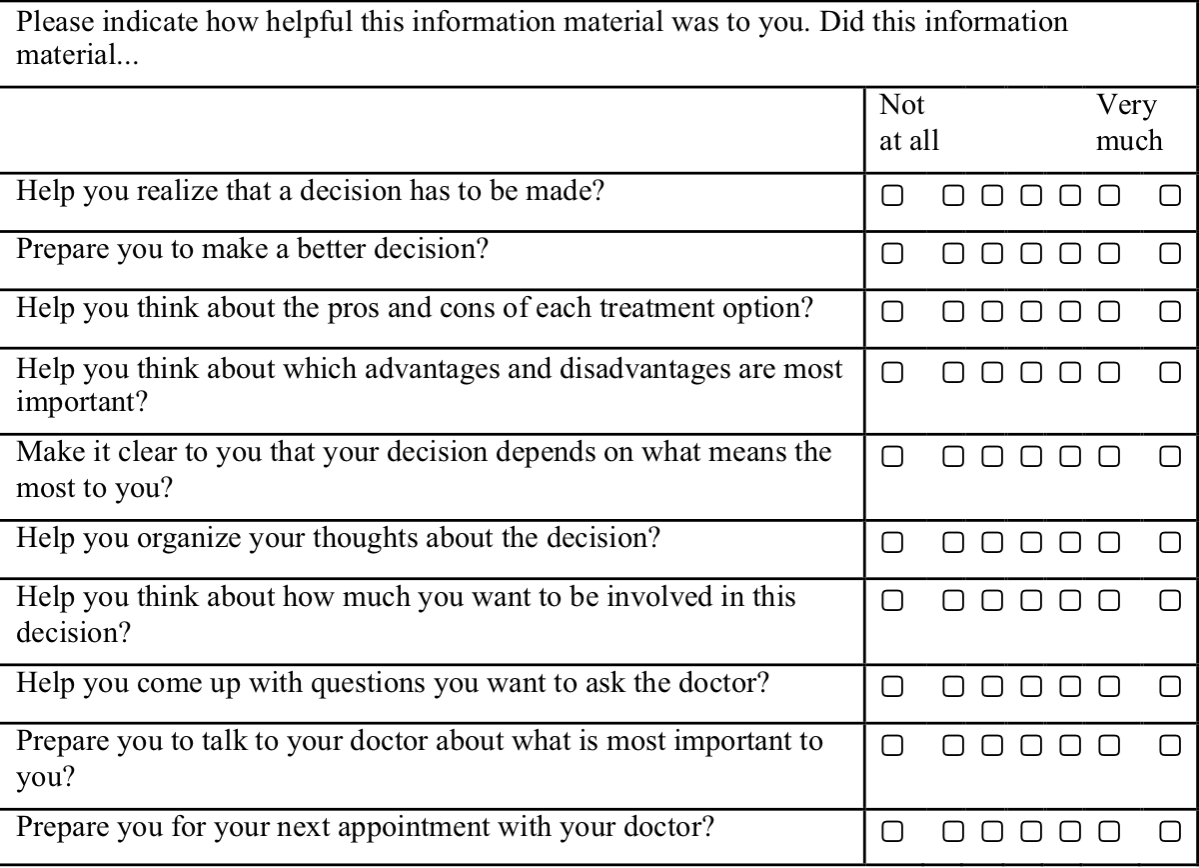
Preparation for decision making scale.

**Figure 3:**
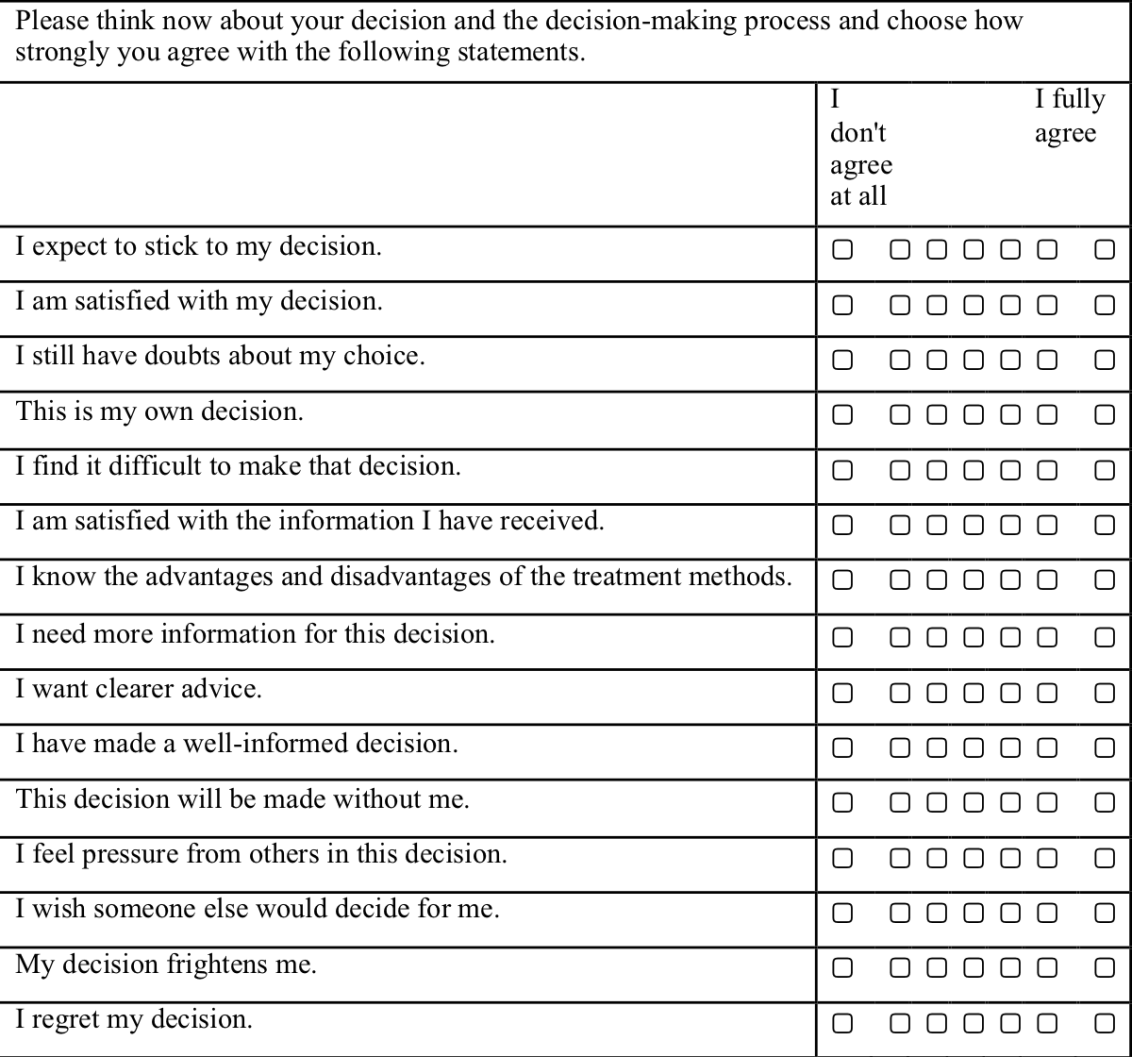
Decision evaluation scale.

A pre-existing decisional conflict questionnaire will be used [38]. Participants will be asked, “How sure are you about your decision for surgical or non-surgical treatment?” and will reply to three statements using a 7-point Likert scale for each: “It's hard for me to make that decision”; “I'm not sure how to act on this decision”; “It's clear which choice is best for me.” They will also be asked “How do you feel about this decision?” and reply to 4 statements using a 7-point Likert scale: “I feel like I made an informed decision”; “My decision shows what is most important to me”; “I expect to stick to my decision”; “I'm satisfied with my decision.” Attitude toward both treatment options will be measured with a 4-item scale (modified following [39]; Figure 1). The knowledge test will be based on the results of Study 1b. The aim will be to provide information on any existing misconceptions or false information regarding the treatment options for Parkinson disease and to test the efficacy of the information provided (a maximum of 10 items). The postintervention preparation for decision making scale is based on [40] (Figure 2). The numerical scale has treatment options ranging from deep brain stimulation on one end of the scale to pure drug therapy on the other end. A decisional conflict questionnaire [38], ratings of feelings about the decision, ratings of attitude toward both treatment options [39], and the decision evaluation scale [41] (Figure 3) will be used.

A maximum of 16 items will be used to evaluate the preferences and motives that were identified in Study 1 with regard to the importance for the decision made such as (the following items are example items only since the items are not yet known): “Please rate the extent to which you agree with the following statements. There is no right or wrong answer. We are only interested in your personal opinion and assessment.”; “The very small, but existing danger of an irreversible side effect (eg, by a brain hemorrhage) with the deep brain stimulation, influenced my decision strongly.”; “The hope for improvement of my independence in everyday life, through deep brain stimulation, has strongly influenced my decision.”; “The concern about psychological impairment (eg, psychosis, states of confusion) in a pure drug treatment had a strong influence on my decision.”

#### Analysis

Data analysis will be performed using SPSS statistical software (version 25; IBM Corp) . We will perform an analysis of variance with posthoc tests (for interval-scaled data) and Mann-Whitney tests (for ordinal-scaled data). We will report all data as means and standard deviations (for interval-scaled data) and the median (for ordinal-scaled data). The level of significance will be set at *P*<.05. Cohen *d* and *r* will be calculated as effect sizes.

### Study 3: Effects of Decision Aids on the Decision-Making Process (Patients With Parkinson Syndrome)

#### Study Design

Based on the decision aids examined in Study 2, the most suitable format for a preference-sensitive decision will be selected and its efficacy will be compared with that of the use of pure factual information. If the need for further modifications of the decision aids becomes apparent in Study 2, we will implement the modifications in collaboration with health professionals. All of the participating patients will be given the opportunity to use the information material of the other condition immediately after the data collection. This ensures that none of the study participants will have any disadvantages, despite the randomized between-subject design.

#### Participants

Recruitment will be carried out with the support of the special outpatient units of the University Hospital Tübingen, neurologists in private practices, and Parkinson self-help groups. The same inclusion and exclusion criteria used in Studies 1a and 1b will be used for Study 3. Since, in this case, only medium to large effect strengths (*d*=.70) have clinical relevance, a sample size of 36 participants per condition will be targeted.

#### Procedure

Basic demographic data (age, sex, education) and the individual perception of the limitations caused by the disease will be collected with the PDQ-39 [34]. Participants will be asked to complete the following questionnaires before and after the use of the decision support: decisional conflict [38]; hypothetical decision (“which treatment option would you probably choose in this situation?”); attitude toward both treatment options (modified following [39]); study-designed knowledge test on risks and side effects of treatment options, mode of action of treatment options, possible advantages and disadvantages of treatment options questionnaires will be used. In addition, the following will be completed after the decision aid has been used: preparation for decision making (based on [40]); evaluation of the preferences and motives identified in Study 1 with regard to the importance for the decision made; decision evaluation scale (based on [41]; reflection on one’s own reasons for the decision; suggestions for improvement (open answer format).

#### Measures

Personal assessment of the current stage and burden of the disease will be measured using the validated questionnaire PDQ-39 [28]. Moreover, the German version of the Apathy Evaluation Scale [35] and the German version of the short-scale for assessing the personality traits in the five-factor model (big five) [36] will be used. In addition, all of the scales in Study 2 will be used. Differences will be only in the introduction to the topic (in Study 3 the participants are patients who are actually affected) and the study design. At the end of the study, the participants will also be given the opportunity to use the information material of the other condition.

#### Analysis

Data analysis will be performed using SPSS statistical software (version 25.0; IBM Corp). We will perform *t* tests (for interval-scaled data) and Mann-Whitney tests (for ordinal-scaled data). We will report all data as means and standard deviations (for interval-scaled data) and as the median (for ordinal-scaled data). The level of significance will be set at *P*<.05. Cohen *d* and *r* will be calculated as effect sizes.

### Data Protection

Personal data will be collected and processed in these studies. For the patients diagnosed with idiopathic Parkinson disease (Studies 1 and 3) the data include their name, sex, age, duration of the disease, other diagnoses, and personal experiences with the disease. In the case of patients, disease data from medical documents (regarding diagnoses and duration of disease) will also be included in the evaluation if necessary. For the medical laypeople (Study 2) the data will include sex, age, and personal experiences with Parkinson disease.

Data will be pseudonymized in a protected electronic database accessible only to authorized staff members, who are bound by professional and data secrecy obligations. In order to verify the correct transfer of the treatment data from the medical file to the encrypted study database, authorized people may inspect the personal disease data related to the study. All employees are bound to secrecy.

The research results from the studies will be published in anonymized form in scientific journals or databases. For the collection, storage, and use of the data, the consent of the participants is required and will be obtained by having them sign the declaration of consent to data protection.

## Results

This study received ethical approval in January 2020. As of June 2020, data collection for Study 1a has begun, and it is estimated that Studies 1a, 1b, 2, and 3 will take approximately 4, 4, 6, and 6 months to complete, respectively. It is planned to present the study results and analyses at international conferences and to submit them to peer-reviewed journals. The study results will additionally be shared with clinicians and patients by presenting them at information events.

## Discussion

This series of studies is intended to shed light on how an evidence-based decision aid for patients with idiopathic Parkinson disease should be designed, in order to facilitate an informed and reflected shared decision-making process. Moreover, the series of studies seeks to contribute to a deeper understanding of individual preferences of patients with idiopathic Parkinson disease and the impact of those preferences on treatment decisions. It is idiosyncratic that the decision-process for patients with idiopathic Parkinson disease may take a relatively long period of time (possibly several years). Idiopathic Parkinson disease is a slowly progressing neurodegenerative disease that makes it possible to protract any final decision through a series of continuous re-evaluations of the patients’ current status and reassessments of their quality of life. During this prolonged decision period, evidence and preferences may develop gradually.

The outcomes from this series of studies will provide valuable new insights into the potential of decision aids for supporting a reflective and informed decision about idiopathic Parkinson disease treatment, and the studies will also help to discover barriers to making an informed decision. The findings would be directly applicable to clinical situations such as (1) results about what kind of information is especially misleading can help physicians and therapists to focus on these aspects in their consultations; (2) knowledge about common patient needs, wishes, and fears can help to tailor the given information to individuals, and (3) if the decision aids have been shown to support shared decision making, physicians can use the tools as additional support for patients. In this case, the information could potentially be provided before medical consultation as preparation. Physicians could then use the consultation time to respond specifically to patient questions and concerns. It is open for discussion whether the findings of this series of studies could be generalized to other fields and contribute to theory with regard to decision situations where empirical evidence (eg, potential superiority of an invasive therapy) and personal preferences (eg, avoidance of surgical intervention) contradict each other.

The strengths of this series of studies are the combination of research methods (qualitative and quantitative methods), the combination of study settings (field and laboratory studies), the adherence to the Revised Program Theory of shared decision making, and the combination of study populations (patients and healthy participants). These strengths will help to produce a broad and more complete view of decision aids for patients with idiopathic Parkinson disease as well as a deeper insight into principal underlying processes of decision-making.

There are limitations to our studies. One issue is that the significance of any potential findings is restricted to patients who suffer from idiopathic Parkinson disease and have not been diagnosed with dementia. Since the decision aids will target this population, the findings will not be generalizable for patients with other diagnoses. Our studies will aim to explore the presentation of motives and outcomes regarding shared decision making for patients with idiopathic Parkinson disease, so we will not be able to make recommendations on how to design and implement other decision support systems in different contexts. Nevertheless, the fine-grained participatory procedure applied here for the design and evaluation of decision aids can also be used for designing decision aids in other contexts or systems. An indepth analysis of patient state of knowledge, needs, wishes, and fears before designing a support system would streamline the design process.

## Abbreviations

PDQ: Parkinson Disease Questionnaire
